# Beetroot juice, exercise, and cardiovascular function in women planning to conceive

**DOI:** 10.1097/HJH.0000000000003562

**Published:** 2023-09-19

**Authors:** Mubarak M.A. Osman, Edward Mullins, Hana Kleprlikova, Ian B. Wilkinson, Christoph Lees

**Affiliations:** a Faculty of Medicine; b Department of Metabolism, Digestion and Reproduction, Imperial College London, London; c The George Institute for Global Health; d Women's Health Research Centre, Queen Charlotte's and Chelsea Hospital, London; e NHS North West London Clinical Commissioning Group, UK; f Department of General Anthropology, Faculty of Humanities, Charles University in Prague, Czechia; g Division of Experimental Medicine and Immunotherapeutics, Department of Medicine, University of Cambridge, Cambridge

**Keywords:** blood pressure, dietary, maternal, monitoring, nitrate, nitric oxide, noninvasive, pre-eclampsia

## Abstract

**Objective:**

Prepregnancy optimization of cardiovascular function may reduce the risk of pre-eclampsia. We aimed to assess the feasibility and effect of preconception cardiovascular monitoring, exercise, and beetroot juice on cardiovascular parameters in women planning to conceive.

**Design and method:**

Prospective single-site, open-label, randomized controlled trial. Thirty-two women, aged 18–45 years, were allocated into one of four arms (1 : 1 : 1 : 1): exercise, beetroot juice, exercise plus beetroot juice and no intervention for 12 weeks. Blood pressure (BP) was measured at home daily. Cardiac output (*CO*) and total peripheral resistance (TPR) were assessed via bio-impedance.

**Results:**

Twenty-nine out of 32 (91%) participants completed the study. Adherence to daily BP and weight measurements were 81% and 78%, respectively (*n* = 29). Eight out of 15 (53%) of participants did not drink all the provided beetroot juice because of forgetfulness and taste. After 12 weeks, exercise was associated with a reduction in standing TPR (−278 ± 0.272 dynes s cm^−5^, *P* < 0.05), and an increase in standing *CO* (+0.88 ± 0.71 l/min, *P* < 0.05). Exercise and beetroot juice together was associated with a reduction in standing DBP ( 7 ± 6 mmHg, *P* < 0.05), and an increase in standing *CO* (+0.49 ± 0.66 l/min, *P* < 0.05). The control group showed a reduction in standing TPR ( 313 ± 387 dynes s cm^−5^) and standing DBP ( 8 ± 5mmHg). All groups gained weight.

**Conclusion:**

Exercise and beetroot juice in combination showed a signal towards improving cardiovascular parameters. The control group showed improvements, indicating that home measurement devices and regular recording of parameters are interventions in themselves. Nevertheless, interventions before pregnancy to improve cardiovascular parameters may alter the occurrence of hypertensive conditions during pregnancy and require further investigation in adequately powered studies.

## INTRODUCTION

Preeclampsia has a worldwide prevalence of 0.2–9.2% [1] and constitutes 10–15% of all direct maternal deaths worldwide [2]. Associated with placental abruption, pulmonary oedema, and thrombocytopenia, pre-eclampsia complications pose a substantial risk to maternal and neonatal wellbeing [3]. Moreover, the disorder's economic burden is substantial, costing in 2012 an estimated $2.18 billion in the United States within the first 12 months of delivery [4]. If the current trend persists, a 322% increase in the rate of severe pre-eclampsia between 1980 and 2010 in the United States, the economic burden will escalate [5] as the prevalence of risk factors for pre-eclampsia (advanced maternal age and obesity) increases [6].

The cause of pre-eclampsia is obscure and is proposed to be because of an interplay of genetic and environmental factors and atypical placentation [7–9]. Such risk factors that play a pivotal role in the development of pre-eclampsia include pregestational diabetes, raised BMI (>30), and prior stillbirth [10]. Additionally, prepregnancy cardiovascular function is a major risk factor in the development of pre-eclampsia and foetal growth restriction (FGR): preconception blood pressure is strongly related to the risk of pre-eclampsia [11]. Furthermore, mean arterial pressure (MAP) and total peripheral resistance (TPR) were higher and cardiac output was lower in healthy women who later developed pre-eclampsia and FGR compared with controls [12]. These genetic and environmental/risk factors are hypothesized to predispose to superficial placentation and inadequate spiral artery remodelling in the first and second trimesters, consequently, leading to a reduction in placental perfusion, which causes an increase in antiangiogenic factors (soluble fms-like tyrosine kinase-1) and other inflammatory mediators [13]. An increase in these mediators causes systemic vascular dysfunction, characterized by an increase in blood pressure and end-organ damage [14].

Treatment options and future development of medications for pre-eclampsia are limited because of anxiety over their potential teratogenic effects [15]. Thus, preventive measures could be a key method of decreasing pre-eclampsia morbidity rates and achieving one of the main targets of the Millennium Developmental Goals: reducing global maternal mortality rates [16]. Aspirin, a recognized prophylactic medication for pre-eclampsia, does not have a primary preventive therapeutic effect but if administered before 16 weeks, it has demonstrated a reduction in preterm pre-eclampsia (<37 weeks) by 62% and early-onset pre-eclampsia (<34 weeks) by 82% [17]. However, the fact that aspirin is a pharmacological substance can discourage mothers from taking the medication [15]. The use of preconception measures, if they were to improve cardiovascular function, could be hypothesized to decrease maternal and perinatal morbidity and mortality at a population level. Furthermore, because of cost-effectiveness [18], simple preventive preconception measures are more accessible than treatments for those from lower income countries where pre-eclampsia mortality rates are much greater [2].

Nonpharmacological alternatives, which have shown promising results as a primary preventive measure in those at risk of cardiovascular disease are beetroot juice (BRJ) and physical exercise [19,20]. Nitrates (NO_3_^−^) are naturally abundant in BRJ [21]. Via the enterosalivary pathway, commensal bacteria convert dietary nitrates into the physiologically active substrate nitric oxide (NO), which plays a pivotal role in normal BP regulation, fetoplacental circulation, and placental angiogenesis [22,23]. By virtue of its ability to improve cardiovascular parameters, exercise is also a potentially effective means of reducing pre-eclampsia risk [20].

Hence, we hypothesized that exercise and BRJ might improve the cardiovascular parameters of healthy women planning pregnancy: in particular reducing blood pressure, total peripheral resistance (TPR), weight, and increasing cardiac output. Moreover, the study aimed to assess satisfaction of the intervention and study protocol and adherence to recording measurements.

## METHODS

### Study design

This feasibility study was a prospective single-site, open-label, randomized control trial (RCT). Participants were recruited via posters, social media, and departmental emails at an inner-city tertiary maternity unit between 09 December 2019 and 02 March 2020. Eligible participants were allocated randomly into one of four arms of the trial (1 : 1 : 1 : 1) for 12 weeks: exercise (EXR), BRJ, EXR&BRJ, and the control (CON) group (no intervention).

Research ethics approval was granted by London Fulham Regional Ethics Committee NHS Health Research Authority and Health Research Authority, IRAS ID:274808. Written informed consent was provided by all participants.

### Participant eligibility

Participant eligibility was assessed by questionnaire. Women aged 18–45 years, contemplating pregnancy in the future, with no health contraindications to moderate vigorous exercise, and employed by the hospital trust were eligible. Exclusion criteria comprised of women presently pregnant, those who planned pregnancy during the study period, or those who became pregnant within the course of the study. For those who withdrew consent or became pregnant during the study period, experimental data were retained up to the point of withdrawal from the study.

### Randomization method

Block randomization, 1 : 1 : 1 : 1 allocation, was performed by a member of staff independent of the research team by assigning participants into a serially numbered, sealed, opaque envelope.

### Study procedure

Participants were scheduled for two clinical assessments: one assessment prior to initiation of the intervention (baseline assessment) and one at study completion at 12 weeks (postintervention assessment). Clinical assessment was completed by a specialist research midwife and a medical student. SBP and DBP were measured by an automatic sphygmomanometer (Microlife Blood Pressure Monitor BPA1 Easy; Cambridge, UK). Measurements were obtained twice standing. A standardized protocol for BP was used for each participant: the nondominant arm was utilized for all measurements with an appropriately sized cuff. Before any cardiovascular assessments, all participants rested for a minimum of 20 min and avoided consuming any caffeinated drinks for 4 h leading up to the blood pressure evaluation. Mean standing BP values were recorded.

Height without shoes was measured using a wall-mounted stadiometer. Weight was examined using a digital scale (Salter Toughened Glass Compact Electronic Bathroom Scale; Kent, UK).

A noninvasive, continuous, whole-body bioimpedance system (NICaS; NI Medical; Hod-Hasharon, Israel) was used to measure *CO* and TPR; four measurements were obtained for each of these cardiovascular parameters in standing position and the mean value was recorded.

Following the clinical assessments, participants were requested to complete a 7-day international physical activity questionnaire (IPAQ). IPAQ scoring protocol was utilized to calculate the Metabolic Equivalent Task Minutes (MET-minutes). All assessments were repeated 12 weeks later to assess for differences.

As per the study arm, participants were provided with a 12-week supply of 70 ml of BRJ supplementation drink (James White Drinks Ltd; Ipswich, UK) containing ∼400 mg nitrate, and consumed the drink each morning with breakfast. Participants were requested to continue their usual diet over the duration of the trial. Two initial personal training consultations were undertaken to construct a personalized resistance and endurance exercise regimen for the participants. Monthly personal training sessions for the next 2 months were provided to those in the exercise arms of the trial. All participants were provided with a home BP monitor (Mircolife Blood Pressure Monitor BPA1 Easy; Cambridge, UK) to measure SBP and DBP once daily. All participants were provided with a notebook to record the following parameters daily for 12 weeks: SBP, DBP, and weight. Furthermore, all participants were contacted fortnightly to assess adherence, answer any queries, and assess willingness to continue with the study. A questionnaire at the end of the study was distributed to participants to identify potential avenues of improvement and assess for the acceptability of the interventions and methods of measurement.

### Outcomes

The primary outcomes of the study were feasibility and adherence to recording key study variables. Feasibility was based on satisfaction with the interventions and study protocol, participant retention, and values obtained from questionnaire responses. Adherence to recording study variables was defined as the number of days a particular variable was recorded (BP or weight) divided by the total number of days in the trial. Data not recorded into the notebook were considered days in which no measurements were undertaken. Secondary outcomes were changes in SBP, DBP, *CO*, TPR, weight, and BMI.

### Sample size calculation

No power calculations were performed because of this being a feasibility study. The National Institute for Health Research (NIHR) advocate that a sample size of 24–50 is adequate for a feasibility study [24–26]. For a study of 32 participants, we anticipated six to seven participants would be lost to follow-up.

### Statistical analysis

Shapiro Wilks test was used to assess the normality of the data. One-way ANOVA with Bonferroni correction or Kruskal–Wallis with Conover's test was used to assess for statistical difference between groups, as applicable. One-tailed Student's *t* test or one-tailed Wilcoxon signed rank-test was used to assess for statistical difference between baseline and postintervention, as applicable. If *P* less than 0.05, the difference was considered to be statistically significant. All statistical analysis was performed using SPSS version 26.0 (IBM Corp., Armonk, New York, USA). Questionnaire responses were collated using NVivo v.12 software. Responses were subjected to thematic analysis; Grounded theory was used for qualitative analysis [27].

## RESULTS

**FIGURE 1:**
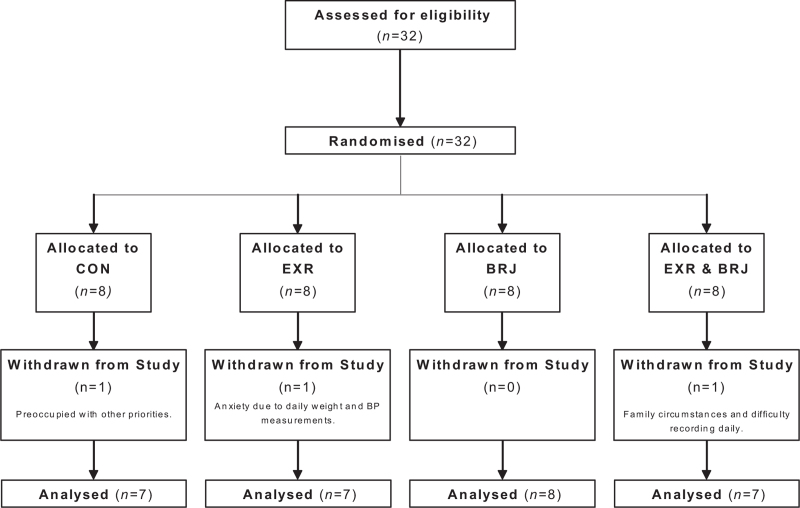
Consolidated standards of reporting trials flow chart of the study. BRJ, beetroot juice; CON, control; EXR, exercise.

Of 32 women eligible, all were enrolled and randomized. Of those enrolled, three withdrew postrandomization and returned no data (29/32). Ninety percent of participants completed the study. Withdrawal rates were similar across all randomized groups: CON, EXR, BRJ, and EXR&BRJ groups were 3.1, 3.1, 0, and 3.1%, respectively. Reasons cited for withdrawal of participation included anxiety because of recording BP and weight daily, preoccupation with personal circumstances, difficulties in recording daily measurements, and complications in scheduling with the personal trainer (Fig. 1). No adverse effects were reported nor side effects such as beeturia and faecal discolouration.

### Efficacy of the interventions

#### Clinic-based measurements

There were no differences in the baseline clinical characteristics, activity score, and cardiovascular parameters between groups (Tables 1 and 2). Moreover, there were no differences between groups for postintervention clinical parameters and change between clinical assessments for all parameters (Table 2). Participants in all groups had an increase in BMI and weight within the 12-week trial period (*P* < 0.05). None of the groups showed any change in the MET-minutes between baseline and follow-up assessments (*P* > 0.05).

**TABLE 1:** Baseline clinical characteristics and demographics stratified by intervention allocation

Clinical Characteristics	CON (*n* = 7)	EXR (*n* = 7)	BRJ (*n* = 8)	EXR&BRJ (*n* = 7)
Age (years)	32 ± 3	32 ± 4	30 ± 6	33 ± 9
Height (cm)	164.1 ± 8.4	165.7 ± 7.2	167.8 ± 8.5	165.7 ± 8.1
Weight (kg)	72.6 ± 18.2	71.7 ± 15.8	69.6 ± 12.3	67.6 ± 15.4
BMI (kg/m^2^)	26.9 ± 6.0	25.9 ± 3.5	24.7 ± 4.1	24.6 ± 6.2
Ethnicity [*n* (%)]				
White	5 (71.4%)	7 (100%)	5 (62.5%)	4 (57.1%)
Black	0 (0%)	0 (0%)	0 (0%)	1 (14.3%)
Asian	1 (14.3%)	0 (0%)	1 (12.5%)	2 (28.6%)
Other	1 (14.3%)	0 (0%)	2 (25%)	0 (0%)

*n* = 29. Values are presented as mean ± SD and *n* (%). Baseline differences between groups were analysed using one-way ANOVA with Bonferroni correction or Kruskal–Wallis with Conover's test and chi-square test, as appropriate. Significant difference was indicated by an ^∗^*P* less than 0.05. BRJ, beetroot juice; CON, control; EXR, exercise.

**TABLE 2:** Characteristics of participant's body composition, activity levels, and cardiovascular parameters before and after the 12-week intervention period

Clinical characteristics	CON (*n* = 7)	EXR (*n* = 7)	BRJ (*n* = 8)	EXR&BRJ (*n* = 7)
Weight (kg)				
Baseline assessment	72.6 ± 18.2	71.7 ± 15.8	69.6 ± 12.3	67.6 ± 15.4
Postintervention assessment	74.2 ± 17.7^∗^	73.5 ± 15.8^∗^	70.7 ± 12.8^∗^	68.6 ± 14.9^∗^
Δ	1.6 ± 1.0	1.8 ± 1.7	1.2 ± 1.5	1.0 ± 1.4
BMI (kg/m^2^)				
Baseline assessment	26.9 ± 6.0	25.9 ± 3.5	24.7 ± 4.1	24.6 ± 6.2
Postintervention assessment	27.6 ± 6.0^∗^	26.5 ± 3.5^∗^	25.2 ± 4.4^∗^	25.1 ± 6.1^∗^
Δ	0.6 ± 0.4	0.7 ± 0.6	0.4 ± 0.5	0.4 ± 0.5
Activity score (Met-min/week); median (range)				
Baseline assessment	1980 (654-7970)	2316 (1291–7760)	1886 (1172–5265)	2232 (1253–4586)
Postintervention assessment	1950 (490–4756)	2430 (1116–9824)	1476 (615–6132)	2250 (882–19492)
Δ (Median)	−30 (−3212 to 1275)	343 (−5346 to 7508)	−1271 (−2164 to 3435)	−725 (−2336 to 18239)
Standing SBP (mmHg)				
Baseline assessment	112 ± 9	110 ± 12	118 ± 18	108 ± 12
Postintervention assessment	112 ± 10	116 ± 18	121 ± 10	112 ± 12
Δ	0 ± 10	5 ± 19	3 ± 19	4 ± 11
Standing DBP (mmHg)				
Baseline assessment	80 ± 6	80 ± 8	79 ± 4	76 ± 9
Posttntervention assessment	73 ± 6^∗^	77 ± 14	77 ± 8	69 ± 4^∗^
Δ	−8 ± 5	−3 ± 10	−2 ± 11	−7 ± 6
Standing *CO* (l/min)				
Baseline assessment	4.95 ± 1.78	4.39 ± 0.64	4.38 ± 0.92	4.15 ± 0.94
Postintervention assessment	5.88 ± 2.52	5.27 ± 1.10^∗^	4.49 ± 1.65	4.64 ± 0.83^∗^
Δ	0.93 ± 1.97	0.88 ± 0.71	0.11 ± 1.43	0.49 ± 0.66
Standing TPR (dynes.s.cm^−5^)				
Baseline assessment	1642 ± 499	1660 ± 196	1757 ± 407	1786 ± 410
Postintervention assessment	1329 ± 466^∗^	1382 ± 298^∗^	1847 ± 654	1489 ± 303
Δ	−313 ± 387	−278 ± 272	90 ± 640	−297 ± 432

Values are presented as mean ± SD, unless stated otherwise. Baseline differences between groups were analysed using one-way ANOVA with Bonferroni correction or Kruskal–Wallis with Conover's test, as appropriate. One-tailed Student *t* test or one-tailed Wilcoxon signed rank-test was used, as appropriate, to assess for significant difference between first and second clinical assessment. Significant difference between assessments was indicated by ^∗^*P* < 0.05. Significant difference between intervention groups was indicated by an ^^^*P* < 0.05. BRJ, beetroot juice; *CO*, cardiac output; CON, control; EXR, exercise; HR, heart rate; MET-min, Metabolic Equivalent Task minutes; TPR, total peripheral resistance. Δ represents the change between the second and first clinical assessment.

Standing SBP showed no difference between baseline and follow-up visits in all groups (Table 2).

A reduction in standing DBP of −7 ± 6mmHg was observed in the EXR&BRJ group (Table 2) (*P* < 0.05) and in the CON group (−8 ± 5 mmHg) (*P* < 0.05).

The EXR&BRJ group and the EXR group showed an increase of +0.49 ± 0.66 l/min (*P* < 0.05) and (+0.88 ± 0.71 l/min) (*P* < 0.05), respectively, for standing *CO*.

The EXR group showed a decrease of −278 ± 272 dynes s cm^−5^ (*P* < 0.05) for standing TPR. BRJ and EXR&BRJ showed no change (*P* > 0.05) for TPR whilst the CON group had a decrease of −313 ± 387 dynes s cm^−5^ (*P* < 0.05).

#### Home-based measurements

When home BP measurements were recorded daily over a 12-week period, there was a reduction in SBP in the EXR and BRJ groups (Supplemental Figure 1). The Pearson's correlation coefficients for the groups were −0.2332 (*P* *=* 0.0126) and −0.3495 (*P* = 0.0002), respectively. However, when these interventions were taken together, the EXR&BRJ group showed no change in SBP (*P* = 0.2063). In the CON group, there was an increase in SBP (*r* = 0.2061, *P* = 0.0257).

In (Supplemental Figure 2) EXR and BRJ groups, DBP decreased, with a Pearson's correlation coefficient of r = −0.6573 (*P* < 0.0001) and *r* = −0.4457 (*P* < 0.0001), respectively. Similar to daily SBP (Supplemental Figure 1d), when these interventions were taken together (EXR&BRJ group), no change was observed (*P* = 0.3695) (Supplemental Figure 2d). A decrease in SBP was observed in the CON group (*r* = −0.2010, *P* = 0.0267) (Supplemental Figure 2a).

### Study acceptability

**FIGURE 2:**
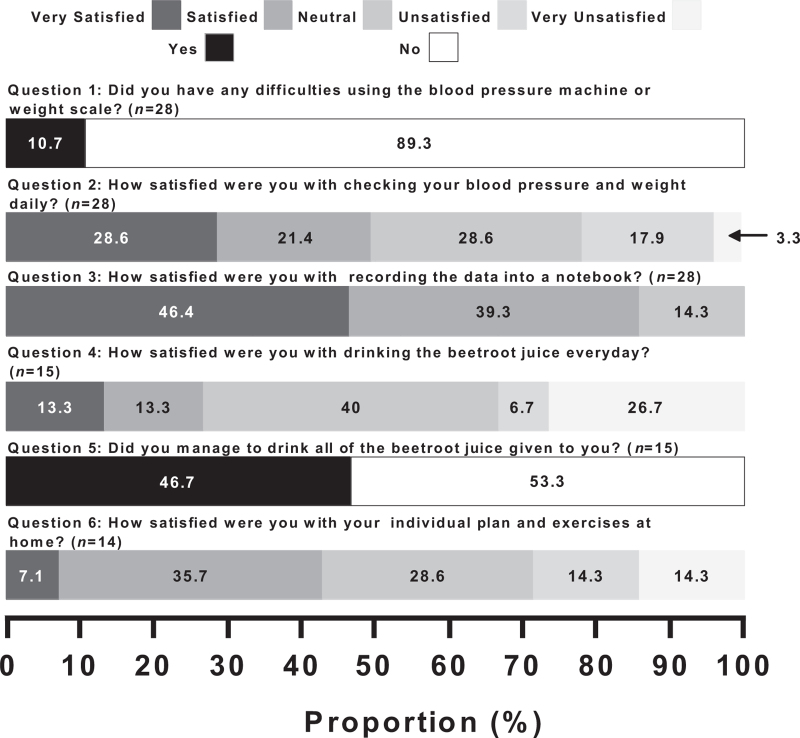
Responses from a questionnaire survey to assess participant acceptability and satisfaction in different aspects of the study.

Twenty-eight of 29 (97%) of participants who completed the full trial, completed the acceptability questionnaire (Fig. 2). Key themes identified from the questionnaire responses were: excessive BP and weight measurements, forgetfulness, lack of personalization of exercise plans, and taste.

Most participants, 25 of 28 (89%), reported no difficulties using the home measuring equipment (Fig. 2, Question 1). However, the daily utilization of the equipment was less acceptable to participants: with six of 28 (21%) being unsatisfied or very unsatisfied with the daily measurements, and 14 of 28 (50%) feeling satisfied or very satisfied (Fig. 2, Question 2). Difficulties in measuring BP and weight daily was recurrently mentioned throughout the transcripts, obtained from open-ended questions in the questionnaire.

Although measuring daily proved difficult for some, participants reported very high levels of satisfaction with recording data into a notebook. Evidenced by 24 of 28 (86%) participants being very satisfied or satisfied, and no participants expressing any form of dissatisfaction (Fig. 2, Question 3). A measurement that encompasses both ease of daily measurements and ease of recording data into a notebook is adherence to recording measurements. Participants often stated that they forgot to record data into their notebooks or were preoccupied with other priorities.

Five out of 15 participants (33.3%) were unsatisfied or very unsatisfied with consuming BRJ daily, compared with 4 of 15 (26.6%) satisfied or very satisfied (Fig. 2, Question 4). Moreover, the majority of participants did not drink all of the BRJ provided to them [8/15 (53.3%)] (Fig. 2, Question 4). An emergent theme was that noncompliance was attributed to forgetfulness and taste.

Similar to the satisfaction with consuming BRJ daily, exercise plans prescribed to participants had a significant proportion of unsatisfied or very unsatisfied participants four of 14 (28.6%). However, six of 14 (42.9%) participants were satisfied or very satisfied (Fig. 2, Question 5). The individual plans, which lacked satisfaction often were not personalized enough and did not cater for the participant's individual circumstances.

When asked about what other aspects of the intervention could be improved, modifications to the protocol to account for variability were often stated in the questionnaire.

### Measurement adherence

Adherence to daily BP and weight measurements were 80.2, and 77.9%, respectively (*n* = 29).

## DISCUSSION

Exercise and BRJ are feasible interventions and women are willing to engage with preconception health monitoring at home with wearable devices and simple clinical measurements. The daily recording of measurements was generally accepted among participants, although less frequent observations, especially body weight, are likely to be more acceptable. The study dropout rate was, at just under 10%, lower than expected and the reasons for withdrawals provide useful information for designing future studies. Daily recording garnered mixed satisfaction with more than one-fifth of participants stating they were unsatisfied or very unsatisfied. This was evidenced by the adherence rate for weight and BP measurements of just above three quarters. Anxiety caused by measuring weight and BP daily could be alleviated by measuring body weight weekly and BP two to three times per week.

Another factor that must be considered in future studies is the acceptability of BRJ. More than one-third of participants were unsatisfied or very dissatisfied with drinking BRJ every day and over half did not drink it at all. This has consequences on the validity of the trial as it has been demonstrated that a 1-week washout period from BRJ can reverse BP changes [28]. Future studies should consider whether there are more palatable ways to prepare nitrate-based supplementation. The acceptability of the EXR intervention also needs further consideration. More than one-quarter of participants were unsatisfied or very unsatisfied with their individual plan and exercises: a recurrent theme was that these were not personalised enough.

Previous studies on healthy, young participants in a clinical setting demonstrate a reduction in SBP and DBP when administered BRJ [28,29–31]. In a phase 2, randomized, double-blind study, dietary nitrate reduced BP in a sustained manner in hypertensive nonpregnant volunteers (8.1/3.8 mmHg) [32]. BRJ alone demonstrated no changes to cardiovascular parameters in this study, most probably because of a lack of statistical power and cannot, therefore, refute that BRJ reduces TPR [33]. Of note, 12 weeks of monitoring alone in controls, EXR, BRJ, and particularly EXR&BRJ were associated with improvements in various cardiovascular parameters in healthy women planning pregnancy. This suggests that preconception health monitoring with lifestyle and nonpharmacological interventions are feasible and potentially effective community-based primary preventive interventions for pre-eclampsia.

An unexpected finding of this study was that our participants in the EXR groups had no significant increase in the MET-min/week. Consistent with this finding is that EXR for 12 weeks did not reduce blood pressure. However, the trial was conducted before, during, and after the third UK pandemic lockdown when gyms were closed and face to face training could not take place. This affected the validity of the exercise intervention as an increase in weight and BMI was seen in all study arms. A systematic review and meta-analysis reported that 11.1–72.4% of individuals had an increase in BMI during lockdown [34]. Thus, it is difficult to ascertain whether the interventions would have had different effects under more normal circumstances.

In a meta-analysis of 11 RCTs of exercise, mean reductions of 2.2 mmHg for SBP and 3.3 mmHg for DBP were reported in normotensive patients [35]. EXR has been demonstrated to reduce the risk of hypertension via different mechanisms including a reduction in vascular resistance, arterial stiffness, and psychological stress [36] and EXR training increases cardiomyocyte contractility and myofilament responsiveness to Ca^2^[37]. Although standing *CO* and TPR were improved, the CON group also showed a significant reduction in TPR. This change from baseline in the control group most likely reflects the fact that there was an intervention in all groups: notably daily home BP recording, home weighing and exercise recording, which could explain behaviour change in the control group.

This is the first study to investigate the synergistic effects of both EXR&BRJ on BP, *CO*, and TPR over an extended period. Improvements in EXR&BRJ were observed for DBP and *CO*. If this is a true finding, an explanation for the greater efficacy of EXR&BRJ can be because of the synergistic effects of both interventions. The increase in NO bioavailability observed after consuming BRJ has also been observed after exercise [38]. Ten weeks of exercise has been shown to upregulate *eNOS* gene expression [39]. This upregulation has also been observed in pregnant women who undertook regular exercise training for 12 weeks associated with increased NO production and a decrease in reactive oxygen species generation in the placenta [40].

There are several limitations to this study. Firstly, as this was a pilot study, no *a priori* sample size calculation was undertaken, hence the study was likely not powered adequately to show true changes in cardiovascular parameters. This provides as an explanation for why the CON group, in whom there was a monitoring intervention also showed signs of cardiovascular improvement. Secondly, intention-to-treat analysis was not possible because of difficulties in obtaining the data of participants who withdrew. Thirdly, whilst DBP and SBP significantly reduced over the 12-week study course using home monitoring devices, this was not mirrored in the baseline and postintervention clinical assessments. This discrepancy probably reflects the additional granularity and statistical power that multiple home measurements over time can provide. Fourthly, we were unable to ascertain exactly how many doses of BRJ were consumed in the group who omitted doses. This presents a challenge in linking any blood pressure alterations to the impact of nitrates; the same limitation can also be applied to the exercise groups. Finally, some data were self-reported, leaving the study susceptible to reporting bias. To minimize this in future studies, memory-based BP monitoring machines could be utilized. Nevertheless, the strength of the study was the low dropout rate in all arms suggesting that it was feasible to assess multiple cardiovascular parameters.

The results of this pilot study show that BRJ and EXR are feasible and well tolerated interventions in potentially modifying cardiovascular parameters in women planning to conceive. The findings support an appropriately powered study on nonpharmacological and home-based monitoring interventions on pregnancy outcomes relating to gestational hypertension. We are careful not to over-interpret a signal in cardiovascular efficacy from EXR&BRJ in the light of the evident drawbacks of the study design and execution: an exercise intervention made much more difficult by lockdown, an underpowered study, and many participants finding BRJ unpalatable. However, this is potentially important as cardiovascular function, in particular low *CO* prior to pregnancy, is associated with pre-eclampsia [25] and there is no intervention that is known to reduce the risk prior to pregnancy.

## ACKNOWLEDGEMENTS

C.C.L. and E.M. are supported by the NIHR Biomedical Research Centre (BRC) based at Imperial College Healthcare NHS Trust and Imperial College London.

We thank Dr Carmel McEniery for her comments on the manuscript.

Sources of funding: C.C.L. and E.M. are supported by the National Institute for Health Research (NIHR) Biomedical Research Centre based at Imperial College Healthcare NHS Trust and Imperial College London. The views expressed are those of the author(s) and not necessarily those of the NHS, the NIHR or the Department of Health.

### Conflicts of interest

There are no conflicts of interest.

## Supplementary Material
