# Supplementary material for: Beetroot juice, exercise, and cardiovascular function in women planning to conceive
Source: J Hypertens. 2023 Sep 19;42(1):101–8. doi: 10.1097/HJH.0000000000003562 (PMC10713001; doi:10.1097/HJH.0000000000003562)
Supplement: Supplementary file 2 [file jhype-42-101-s002.ppt]

## Slide 1
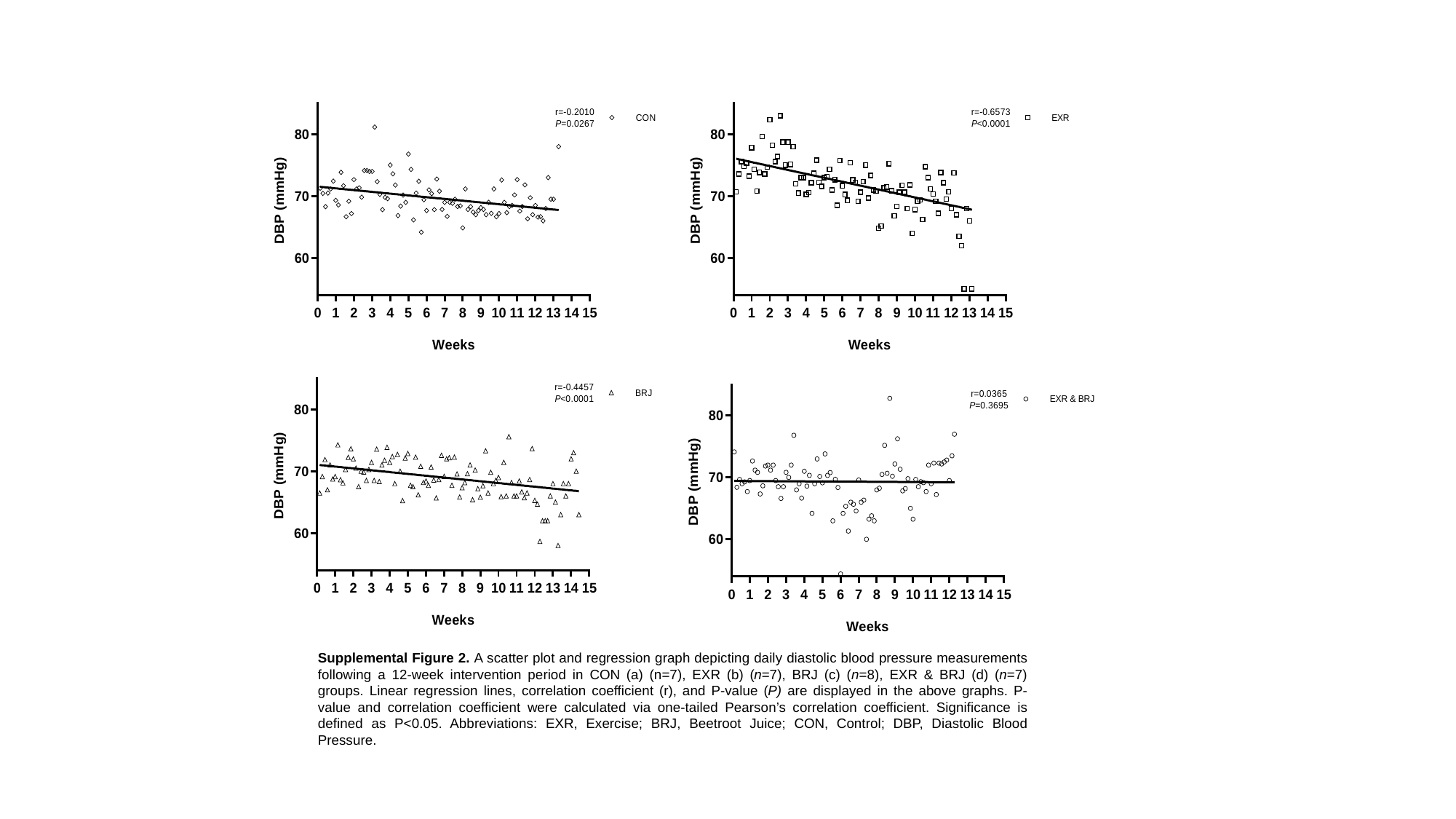

Supplemental Figure 2. A scatter plot and regression graph depicting daily diastolic blood pressure measurements following a 12-week intervention period in CON (a) (n=7), EXR (b) (n=7), BRJ (c) (n=8), EXR & BRJ (d) (n=7) groups. Linear regression lines, correlation coefficient (r), and P-value (P) are displayed in the above graphs. P-value and correlation coefficient were calculated via one-tailed Pearson’s correlation coefficient. Significance is defined as P<0.05. Abbreviations: EXR, Exercise; BRJ, Beetroot Juice; CON, Control; DBP, Diastolic Blood Pressure.
